# Anaphylactic Reaction to Protamine Sulfate: A Case Report

**DOI:** 10.7759/cureus.69456

**Published:** 2024-09-15

**Authors:** Ana David do Carmo, Margarida L Nascimento, Inês R Carvalho, André Rosa Alexandre, Ricardo Arruda Pereira

**Affiliations:** 1 Internal Medicine Department, Hospital da Luz Lisboa, Lisbon, PRT; 2 Endocrinology Department, Hospital da Luz Lisboa, Lisbon, PRT; 3 Intensive Care Department, Hospital da Luz Lisboa, Lisbon, PRT; 4 Cardiothoracic Surgery Department, Hospital da Luz Lisboa, Lisbon, PRT

**Keywords:** anaphylactic shock, cardiothoracic surgery, heparin reversal, protaminated insulin, protamine sulfate

## Abstract

Protamine sulfate is a standard agent used to reverse heparin anticoagulation during cardiovascular procedures such as coronary artery bypass grafting (CABG). Although rare, it can be associated with severe adverse reactions.

We present a case of a 68-year-old male with type 2 diabetes, who was on insulin lispro protamine and underwent elective CABG. Shortly after protamine administration to reverse heparin during extracorporeal circulation (ECC), the patient experienced anaphylactic shock, manifesting as tachycardia, hypotension, facial flushing, periorbital edema, and elevated serum tryptase levels. Despite prompt treatment with volume resuscitation, epinephrine, and corticosteroids, the patient’s condition remained unstable, necessitating a second period of ECC for additional bypass grafting.

The case contributes to the clinical literature by providing an illustrative example of how protamine hypersensitivity can complicate postoperative recovery, emphasizing the importance of vigilance and comprehensive management strategies in preventing and addressing such reactions.

## Introduction

Protamine sulfate is a polypeptide derived from salmon sperm and is primarily used to reverse heparin-induced anticoagulation during cardiovascular surgeries such as coronary artery bypass grafting (CABG) [1-3]. When administered intravenously, protamine neutralizes heparin by forming a neutral salt, effectively reversing anticoagulant effects. Additionally, protamine is used in neutral protamine Hagedorn (NPH) insulin and premixed insulin preparations to prolong insulin absorption and action [3,4].

Despite its routine use, protamine can cause adverse effects ranging from vasodilation with transient systemic hypotension (related to rapid drug administration) to life-threatening cardiopulmonary dysfunction and shock [1,3]. Although life-threatening protamine reactions are rare, occurring in approximately 2.6% of cardiac surgeries, patients with previous exposure to protamine-containing products, such as insulin, are at an increased risk of hypersensitivity reactions [1,2].

We report a case of a diabetic patient treated with insulin lispro protamine who developed anaphylactic shock after receiving protamine sulfate. It was administered to reverse heparin anticoagulation used during CABG after the patient was taken off extracorporeal circulation (ECC).

This case contributes significantly to the existing literature and clinical practice, particularly in the fields of cardiovascular surgery, anesthesiology, intensive care medicine, and perioperative care. First and foremost, it provides a rare but critical example of protamine-induced anaphylaxis. Although protamine reactions are known, the severity of this case adds valuable insight into the variability and complexity of this condition. The case underscores the need for heightened awareness, prompt diagnosis, and rapid intervention when protamine-induced anaphylaxis occurs.

## Case presentation

A 68-year-old male with a past medical history of type 2 diabetes who had been on insulin lispro protamine suspension for 15 years, hypertension, dyslipidemia, obstructive sleep apnea, and prior smoking was admitted for elective CABG due to severe coronary artery disease. In the months preceding surgery, the patient reported increasing tiredness and dyspnea, and a cardiac stress test revealed ischemia. Coronary catheterization confirmed severe stenoses in the left anterior descending (LAD), left circumflex, and right coronary arteries. The planned surgery involved double bypass grafting under balanced anesthesia with ECC. The surgeon performed a bypass from the left internal mammary artery to the LAD and a saphenous vein graft to the obtuse marginal artery.

Following the successful completion of ECC, protamine sulfate was administered to reverse heparin anticoagulation. Almost immediately, the patient developed tachycardia and hypotension with facial flushing, periorbital edema, and conjunctival erythema. The patient exhibited good peripheral perfusion and warm skin, with no signs of bronchospasm and adequate arterial blood gases.

A first echocardiogram was performed showing no complications, preserved left ventricular systolic function, and no abnormalities in segmental contractility, which made the diagnosis of cardiogenic shock unlikely.

No significant blood loss was reported during the procedure, blood tests showed stable hemoglobin levels, and urine output remained normal, making hypovolemic shock a less likely diagnosis.

The most likely diagnosis was assumed to be anaphylactic shock triggered by protamine sulfate, based on the clinical presentation and the timing of symptom onset. Initial management included intravenous fluids, epinephrine, clemastine, and hydrocortisone. Despite these interventions, the patient continued to exhibit signs of circulatory shock.

The serum tryptase level was elevated at 28.40 µg/L (reference value <11 µg/L), supporting the diagnosis of anaphylactic shock.

Another reevaluation echocardiography revealed severe apex hypokinesis requiring re-entry into ECC. Another bypass with a saphenous vein to LAD was performed. There was a clear improvement in apical contractility after ECC withdrawal, without any segmental changes in contractility (Video 1).

**Video 1:** Transthoracic echocardiogram after extracorporeal circulation exit showing good segmental contractility.

Due to the risk of exacerbating the allergic reaction, protamine was not re-administered to reverse heparin, and the patient was managed with transfusions of two units of packed red blood cells (PRBC), two platelet concentrates (PC), and two fresh frozen plasma (FFP) units. He was transferred to the intensive care unit (ICU) under sedation with propofol, on volume assist-controlled ventilation, and vasopressor support with adrenaline and noradrenaline. Initial lactate levels were elevated at 5.1 mmol/L, suggesting inadequate tissue perfusion despite acceptable peripheral circulation (normal capillary refill time).

In the first 12 hours postoperatively, the patient developed hemorrhagic shock due to significant blood loss (2680 mL) from the chest drains, requiring massive transfusion (six PRBCs, two PCs, two FFP units, and 1500 IU of prothrombin complex concentrate). He remained sedated, intubated, and on vasopressor support for five days.

While in the ICU, there were several complications. First, a left hemothorax complicated by methicillin-resistant *Staphylococcus epidermidis* empyema that needed two video-assisted thoracoscopic surgeries (VATS), one with decortication and the second with debridement. Second, a mediastinitis with mediastinal collections and sternal instability, requiring revision of the sternotomy, drainage, and sternal stabilization. Third, *Enterobacter cloacae* catheter-associated urinary tract infection and methicillin-susceptible *Staphylococcus aureus* hospital-acquired pneumonia. These infectious complications were treated with directed antibiotic therapy. The patient also had episodes of atrial fibrillation with rapid ventricular response demanding administration of amiodarone, carvedilol, and anticoagulation with enoxaparin, as well as acute kidney injury (maximum creatinine = 200 mg/dL) and delirium.

After a prolonged ICU stay, the patient was transferred to the internal medicine department on the 28th postoperative day. He remained hospitalized for another 16 days, under cardiorespiratory and motor rehabilitation, with a very favorable clinical evolution. The NPH insulin was withheld and later replaced by glargine insulin.

The patient was reassessed at a follow-up appointment two weeks after discharge and was asymptomatic, with no new complications reported.

## Discussion

Albeit rare, protamine administration can be associated with severe systemic reactions with considerable morbidity and mortality [4]. A few cases of life-threatening adverse reactions and death during cardiac surgery due to anaphylactic reactions to protamine have already been described in multiple clinical cases before [5-7].

Patients with the potential for prior sensitization to protamine-containing products show an increased risk for protamine allergic reactions [8]. The most common predisposing factor is treatment with protamine-containing insulin in patients with diabetes mellitus [4]. Other risk factors include prior cardiovascular procedures with protamine administration, fish allergy, and vasectomized men since they may develop antibodies to sperm antigens as previously presented by Singh et al.* *[2,8,9].

In this case, we presented a diabetic patient medicated with the premixed insulin lispro protamine, hence he had already been exposed to protamine before the surgical procedure in a similar way to that described by Khan and Pacleb [10].

The initial clinical presentation of tachycardia, hypotension, facial flushing, periorbital edema, and conjunctival erythema, occurring almost immediately after protamine sulfate administration, in this previously sensitized patient, strongly suggested anaphylactic shock. The elevated serum tryptase level further supported this diagnosis, as tryptase is a marker of mast cell degranulation most commonly associated with anaphylaxis.

The treatment of adverse reactions to protamine comprises providing support to the affected organs and mitigating the effects of histamine, but despite volume resuscitation, epinephrine, clemastine, and hydrocortisone, the patient's condition did not stabilize, indicating ongoing circulatory shock.

Although the first intra-operatory echocardiogram revealed preserved left ventricular systolic function and no segmental contractility abnormalities, which initially suggested that cardiogenic shock was unlikely, the subsequent echocardiogram demonstrated severe apex hypokinesis, prompting a re-entry into ECC and the performance of an additional bypass graft. The observed improvement in apical contractility after ECC withdrawal indicated a component of cardiogenic shock, likely secondary to the severe anaphylactic reaction and subsequent myocardial dysfunction.

The absence of significant blood loss during the procedure, stable hemoglobin levels, and normal urine output made hypovolemic shock a less likely diagnosis. Although hypovolemic shock due to bleeding was considered, the patient's hemodynamic stability and lab results did not support this etiology.

It was decided not to readminister protamine after the discontinuation of the second ECC period, considering the risk of exacerbating a protamine reaction outweighed the risks of severe bleeding with spontaneous reversal of heparin.

Subsequently, the patient developed hemorrhagic shock due to significant blood loss from chest drains, requiring massive transfusions and administration of prothrombin complex concentrate. This complication likely arose from coagulopathy in the context of ongoing anticoagulation without adequate protamine reversal.

All the complications that the patient went through at the ICU highlight the challenges of managing complex postoperative patients, especially those with multiple pre-existing conditions. Each issue was addressed through a combination of surgical interventions, targeted antimicrobial therapies, and supportive care, contributing to a favorable recovery outcome.

## Conclusions

Protamine sulfate, though effective and routinely used to reverse heparin anticoagulation in cardiovascular surgeries, can lead to severe systemic reactions in a subset of patients. This case underscores the importance of heightened vigilance, especially in patients with risk factors like prior exposure to protamine-containing products, including certain insulin formulations. Given that there is no alternative to protamine and its use remains necessary in such procedures, identifying patients at higher risk for hypersensitivity reactions is essential. Proactive measures, such as detailed preoperative evaluations, monitoring for early signs of anaphylaxis, and having protocols in place for rapid intervention, can mitigate the risks and improve patient outcomes. Ultimately, better recognition of predisposing factors can allow for timely preparation, swift diagnosis, and optimal management of these rare but potentially life-threatening complications.

## References

[1] Levy JH, Ghadimi K, Kizhakkedathu JN, Iba T (2023). What's fishy about protamine? Clinical use, adverse reactions, and potential alternatives. J Thromb Haemost.

[2] Lee CH, Cheng HC, Ko LW (2013). Successful treatment of anaphylactic shock after protamine administration-report of a case. Emerg Med Open Access.

[3] Panos A, Orrit X, Chevalley C, Kalangos A (2003). Dramatic post-cardiotomy outcome, due to severe anaphylactic reaction to protamine. Eur J Cardiothorac Surg.

[4] Nybo M, Madsen JS (2008). Serious anaphylactic reactions due to protamine sulfate: a systematic literature review. Basic Clin Pharmacol Toxicol.

[5] Chu YQ, Cai LJ, Jiang DC, Jia D, Yan SY, Wang YQ (2010). Allergic shock and death associated with protamine administration in a diabetic patient. Clin Ther.

[6] Valchanov K, Falter F, George S (2019). Three cases of anaphylaxis to protamine: management of anticoagulation reversal. J Cardiothorac Vasc Anesth.

[7] Kudoh O, Warabi K, Yamaguchi K, Ichinose M, Iizuka T, Inada E (2006). A case of anaphylactic shock in an elderly man following protamine sulfate administration during emergent off-pump coronary artery bypass grafting. (Article in Japanese). Masui.

[8] Peeters M, Yilmaz A, Vandekerkhof J, Kaya A (2020). Protamine induced anaphylactic shock after peripheral vascular surgery. Ann Vasc Surg.

[9] Singh V, Song C, Woodbury A (2017). The plight of protamine for heparin reversal in sensitized individuals. Pol Ann Med.

[10] Khan B, Pacleb A (2023). Diabetic patients on NPH insulin with increased risk of developing severe reactions to protamine sulfate. Ann Allergy Asthma Immunol.

